# In vitro synergy of sertraline and tetracycline cannot be reproduced in pigs orally challenged with a tetracycline resistant *Escherichia coli*

**DOI:** 10.1186/s12866-018-1383-5

**Published:** 2019-01-11

**Authors:** Sofie Kromann, Anna Hvidtfeldt, Mette Boye, Dorte Bratbo Sørensen, Steffen Jørgensen, Jens Peter Nielsen, Rikke Heidemann Olsen

**Affiliations:** Department of Veterinary and Animal Sciences, Faculty of Health and Medical Sciences, University of Copenhagen, Frederiksberg, Denmark

**Keywords:** Antimicrobial resistance, Tetracycline, Sertraline, Synergy, Pigs, *E. coli*

## Abstract

**Background:**

Antimicrobial helper-compounds may reverse antimicrobial resistance. Sertraline, a antidepressant drug, has been suggested as a tetracycline helper-compound. Tetracycline is the preferred antimicrobial for treatment of enteric diseases in pigs. This study is the first to evaluate the potency of sertraline as a tetracycline adjuvant in pigs.

**Methods:**

Forty-eight nursery pigs were divided into four treatment groups: Tetracycline, sertraline, tetracycline/sertraline or un-medicated control. Fecal and ileal samples were obtained before treatment, 48 h and nine days after five days of treatment, respectively. Colony forming units (CFU) of tetracycline resistant coliforms in each sample (ileal or fecal) and CFU of an orally inoculated tetracycline-resistant strain of *Escherichia coli* were determined at each sampling point. The microbiome of fecal and ileal and samples was analyzed by sequencing of the 16S V3-V4 region.

**Results:**

The results did not provide evidence that sertraline in combination with tetracycline has any impact on tetracycline resistant bacteria in either fecal or ileum samples, while in the tetracycline treated group of pigs, an increase in the prevalence of a tetracycline resistant indicator strain of *Escherichia coli* shortly after ended five-day treatment was observed. The ileal samples obtained shortly after ended treatment showed treatment-associated changes in the composition of the microbiota in the groups of pigs treated with tetracycline (+/−) sertraline. While tetracycline treatment increased the abundance in the reads of *E. coli*, sertraline/tetracycline treatment led to increased abundances of *Streptococcus* spp. and decreased abundances of *Lactobacillus* spp. However, all observed differences (on CFU counts and microbiota composition) between groups shortly after treatment had diminished in less than two weeks after last treatment day.

**Conclusions:**

Sertraline (+/−) tetracycline treatment did not reduce the long-term level of tetracycline-resistant bacteria in the feces or small intestine contents of piglets compared to the un-medicated control group of pigs. The result of this study reflects the importance of in vivo studies for confirmation of the antimicrobial helper-compound potential of an in vitro active compound.

**Electronic supplementary material:**

The online version of this article (10.1186/s12866-018-1383-5) contains supplementary material, which is available to authorized users.

## Background

For many years tetracyclines have been the most commonly used antimicrobials to treat pigs with enteric disease [1]. The widespread uses of tetracyclines have been suggested as the major cause of the increase in tetracycline resistant bacteria, in particular in pigs [2]. Tetracyclines have been favored in agriculture because of its broad-spectrum activity, low toxicity, many formulations for oral use and the relative low price [3, 4], but the need to bring down the amount of antimicrobials used in the livestock industry is inquisitional to inhibit further emergence of antimicrobial resistance [5, 6]. In addition, antimicrobial helper-compounds have received increased focus. Helper-compounds may be described as drugs that enhance the activity of antibiotics [7]. Some helper-compounds possess antimicrobial activities themselves [7], while others interfere with the resistance mechanism of the bacterial resistance, e.g. by blocking antimicrobial efflux pumps [8]. Sertraline, a selective serotonin reuptake inhibitor (SSRI), is a medical compound normally prescribed for human mental disorders, such as depression and anxiety, and it has been suggested as tetracycline helper-compound by several research groups [9–13]. Although sertraline itself do possess antimicrobial activity against a number of Gram-negative bacterial species, the main reason of interest for sertraline as a helper-compound is due to the interference with the tetracycline resistant efflux pump, TetA [10, 13]. While several in vitro results have been published on the antimicrobial helper-drug activities of SSRIs, including sertraline, only very limited literature on the clinical in vivo effect of sertraline’s helper-compound activity exist.

The aim of the present study was to evaluate the influence of sertraline on the CFU of intestinal commensal tetracycline coliforms in concurrent tetracycline treated piglets compared to the level in non-medicated, sertraline - or only tetracycline treated groups of pigs. For the same groups, the impact of treatment on the ileal microbiota composition was assessed.

## Results

### Level of tetracycline resistant bacteria in feces before inoculation

**Fig. 1:**
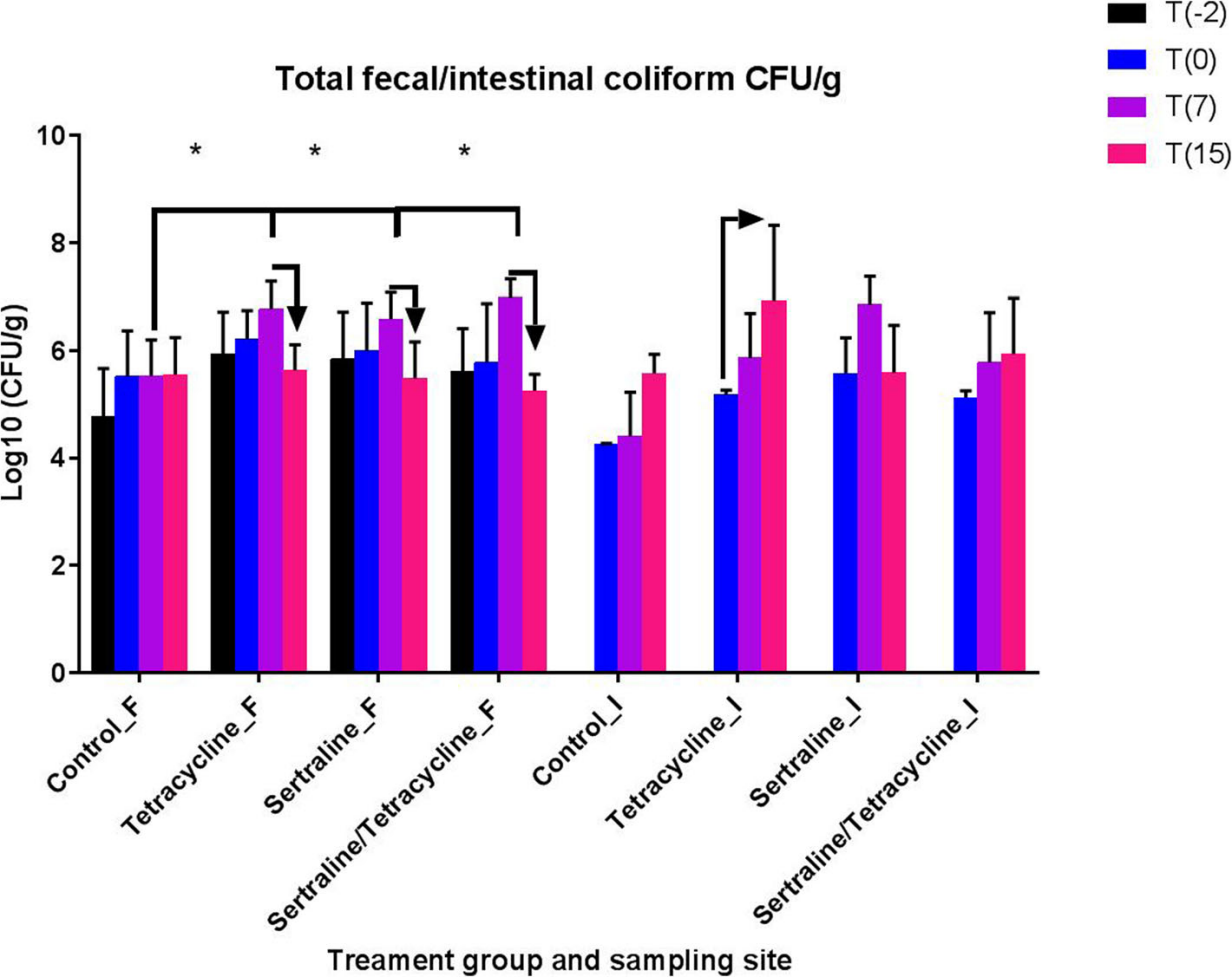
Total fecal (_F) and intestinal (_I) tetracycline resistant CFU Numbers (in log_10_ CFU per gram feces (_F) or ileum (_I)) of total tetracycline resistant CFU at different time points in four different treatment groups. Vertical bars indicate standard deviations. Timepoint T(− 2) refers to two days before treatment start, and just prior to oral inoculation of all pigs with a tetracycline/rifampicin resistant *Escherichia coli* strain. At T(− 2) 12 individual fecal samples were obtained from each group. No pigs were euthanized at this point, therefore no ileum samples were obtained at T(− 2). Time point T(0) refers to the first treatment day. Samples were obtained prior to the first treatment at the same day. From each group 12 individual fecal samples were obtained. Hereafter, two pigs were euthanized at ileum samples were obtained from 2X4 pigs. Time T(7) refers to 48 h after the last of five days of treatment. From each group 10 individual fecal samples were obtained. Hereafter, five pigs were euthanized and ileum samples were obtained from 5X4 pigs. Time T(9) refers to nine days after the last of five days of treatment. From each group five individual fecal samples were obtained. Hereafter, the five pigs in each group were euthanized and ileum samples were obtained from 5X4 pigs. For visual purposes all CFU counts at the different time points has been included in the same figure, statistical analysis were, however, done for each time point, each treatment group at different sampling time and sampling site (fecal or ileum) individually. Asterisks over straight lines indicate a statistical difference at the same time point between treatment groups. Arrows indicate statistical difference at the different time points within each treatment groups. Hash tag indicates that group is statistically different from the other groups at the same sampling time. Statistical significant differences between fecal and ileum samples are not shown on the figure

Two days before treatment start and just prior to inoculation of all pigs in all four groups (Time point T[− 2] the level of fecal tetracycline resistant coliforms in the 48 individual fecal samples ranged from 3.3–7.7 log_10_ CFU per gram of feces (Fig. 1). None of the fecal samples contained bacteria which could be cultured on the indicator agar plate (MacConkey agar supplemented with tetracycline, ampicillin and rifampicin).

### Level of tetracycline resistant bacteria in feces and ileum immediately before treatment start

**Fig. 2:**
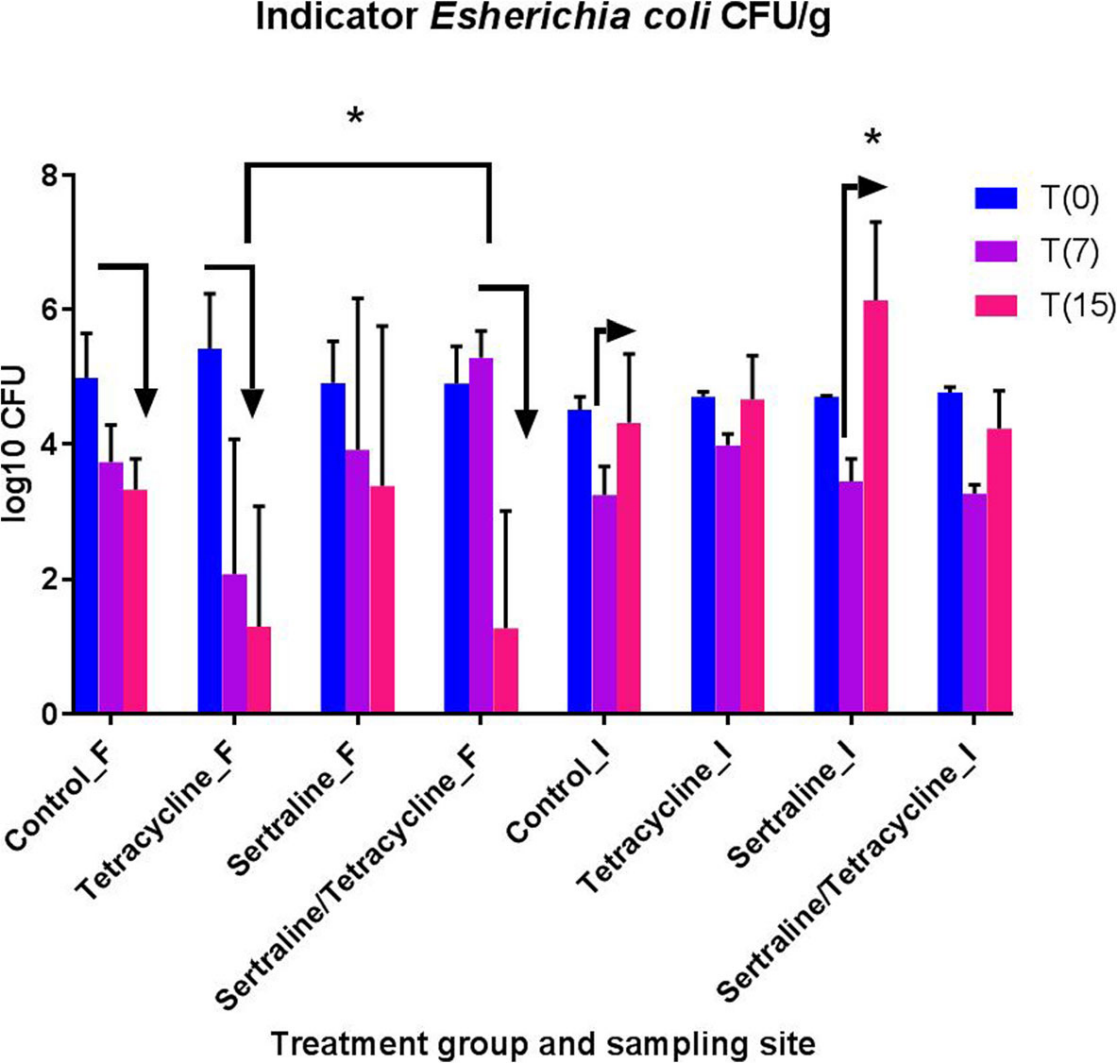
Indicator *E. coli* CFU Numbers (in log_10_ numbers of CFU per gram feces (_F) or ileum (_I)) of an indicator *E. coli* strain at three different time points in four different treatment groups. Vertical bars indicate standard deviations. Before the pigs were inoculated with the indicator strain, no pigs carried a detectable level of the indicator strain in feces (data not shown on the figure). Time point T(0) refers to the first treatment day. Fecal and ileum samples were obtained prior to the first treatment at the same day. From each group 12 individual fecal samples were obtained. Hereafter, two pigs were euthanized and ileum samples were obtained from 2X4 pigs. Time T(7) refers to 48 h after the last of five days of treatment. From each group 10 individual fecal samples were obtained. Hereafter, five pigs were euthanized at ileum samples were obtained from 5X4 pigs. Time T(15) refers to nine days after the last of five days of treatment. From each group five individual fecal samples were obtained. Hereafter, the five pigs in each group were euthanized and ileum samples were obtained from 5X4 pigs. For visual purposes all CFU count at the different time points has been included in the same figure, statistical analysis were, however, done for each time point and sampling site individually. The asterisk indicate that the ileum samples from the tetracycline treated group had a significant higher level than ileum samples from the three other groups at time point T(7)

Two days after inoculation with the indicator bacteria (Time point (T[0]), fecal samples were obtained from all 48 pigs, while ileal samples were obtained from two pigs per treatment group. At T[0] the indicator bacteria could be re-isolated (confirmed by PCR) in all fecal samples, while for one out of eight pigs the indicator bacteria could not be detected in the ileal samples at this time point. The level of the indicator *E. coli* at T[0] in fecal and ileum samples varied between 4.3–6.3 log_10_ to 0–4.1 log_10_ CFU/g, respectively, (Fig. 2). There was no significant difference between the groups in the level of the indicator *E. coli* at T[0] (Fig. 2,). Overall, in paired fecal and ileum samples from the same pig there were lower CFU counts observed in the ileum samples.

### Level of tetracycline resistant bacteria in fecal/ileal samples after treatment

Forty-eight hours after the last of five treatment days, fecal samples were obtained from all pigs in each group (Time point T[7]). Hereafter, five pigs from each group were randomly chosen for euthanization and from these pigs ileum samples were obtained as well. Nine days later, fecal and ileum samples were obtained from the remaining pigs in each group (Time point T[15]).

**Fig. 3:**
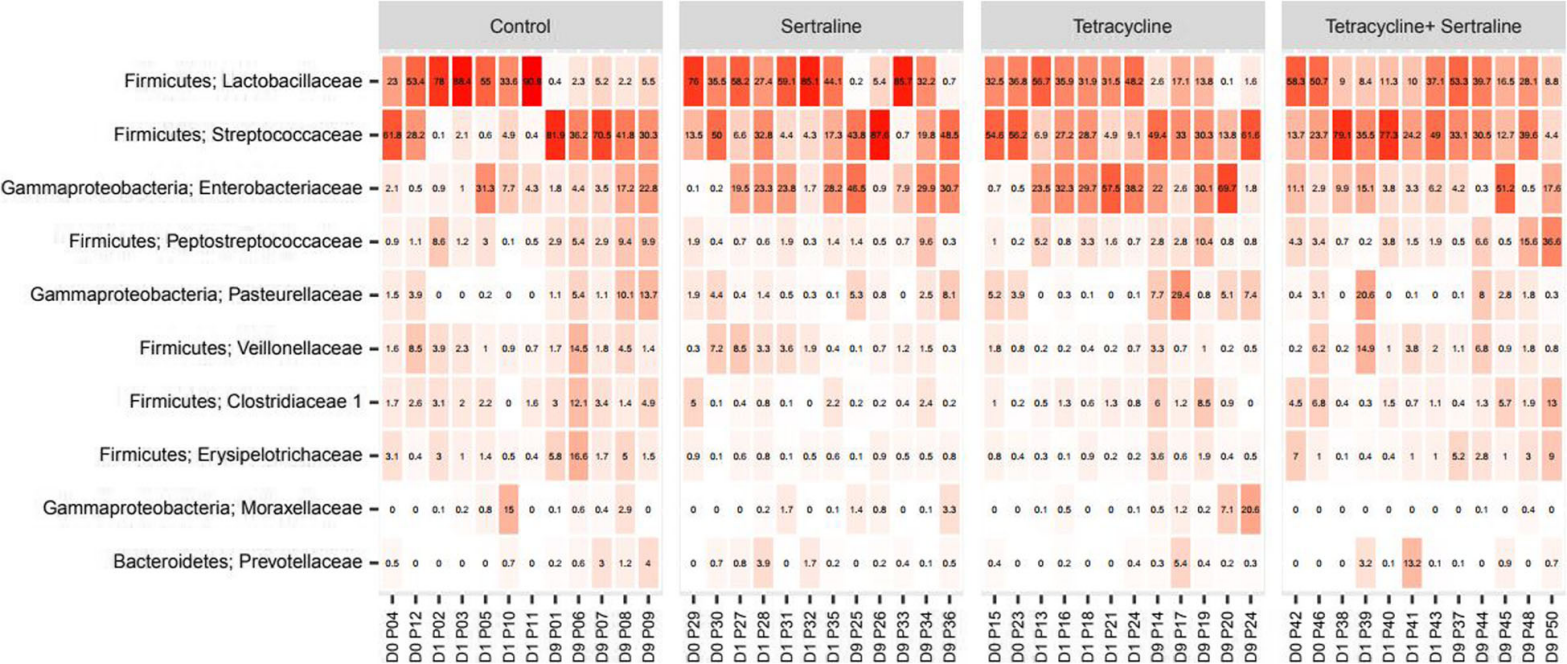
Principle component analysis (PCA) of ileum microbiota composition at time T[0] and T[7] Identification of samples with similar microbiota composition using multivariate statistics (PCA). Each red or blue point represent the microbial community in a specific sample obtained just prior to treatment start (D0/T[0]) or 48 h after last day of treatment (D1/T[7]), respectively

At T[7] there was significantly higher count of tetracycline resistant coliform CFU per gram in fecal samples obtained from tetracycline, sertraline or sertraline/tetracycline treated pigs compared to the CFU per gram fecal samples obtained from un-mediated control pigs (Fig. 1). Similarly, ileum samples from un-mediated control pigs contained significantly lower tetracycline resistant coliform CFU per gram compared to the tetracycline or sertraline treated group at T[7] (Fig. 1).Ileum samples from the tetracycline treated group (Group 2) had a significant higher level of the indicator *E. coli* compared to the level in any of the other three groups (Fig. 3), while there were no difference between the groups in the level of the indicator bacteria in fecal samples (Fig. 2).

At T[15] there was no statistical significant difference in any CFU counts *between* the four groups, whereas there were several difference in CFU counts *within* each of the groups (Fig. 1). There was a significant decrease in total fecal tetracycline resistant coliform CFU per gram at time T[15] compared with T[7] for the tetracycline treated group (Group 2), the sertraline treated group (Group3) and sertraline/tetracycline treated group (Group 4), respectively (Fig. 1), while the were no difference in the level of fecal tetracycline resistant coliform CFU in the control group. The level of the indicator *E. coli* in feces also decreased significantly from time T[0] to time T[15] in the tetracycline and sertraline/tetracycline treated groups (Fig. 2).

### Microbiota sequencing results

Sample preparation and sequencing were successful for 67 out of 68 samples (quality of sample preparation of DNA from a fecal sample from a pig in the tetracycline-treated group obtained nine days post ended treatment did not pass quality control). The number of reads, OTU and Shannon index per sample is stated in Additional file 1: Table S1. The average Shannon index was significantly lower in ileum samples (average 1.78 for all ileum samples combined) then the fecal samples (4.62). There were no significant differences in the Shannon indexes between ileal samples from different treatment groups or different sampling times.

### The impact of treatment and time post treatment on the ileum microbiota composition

**Fig. 4:**
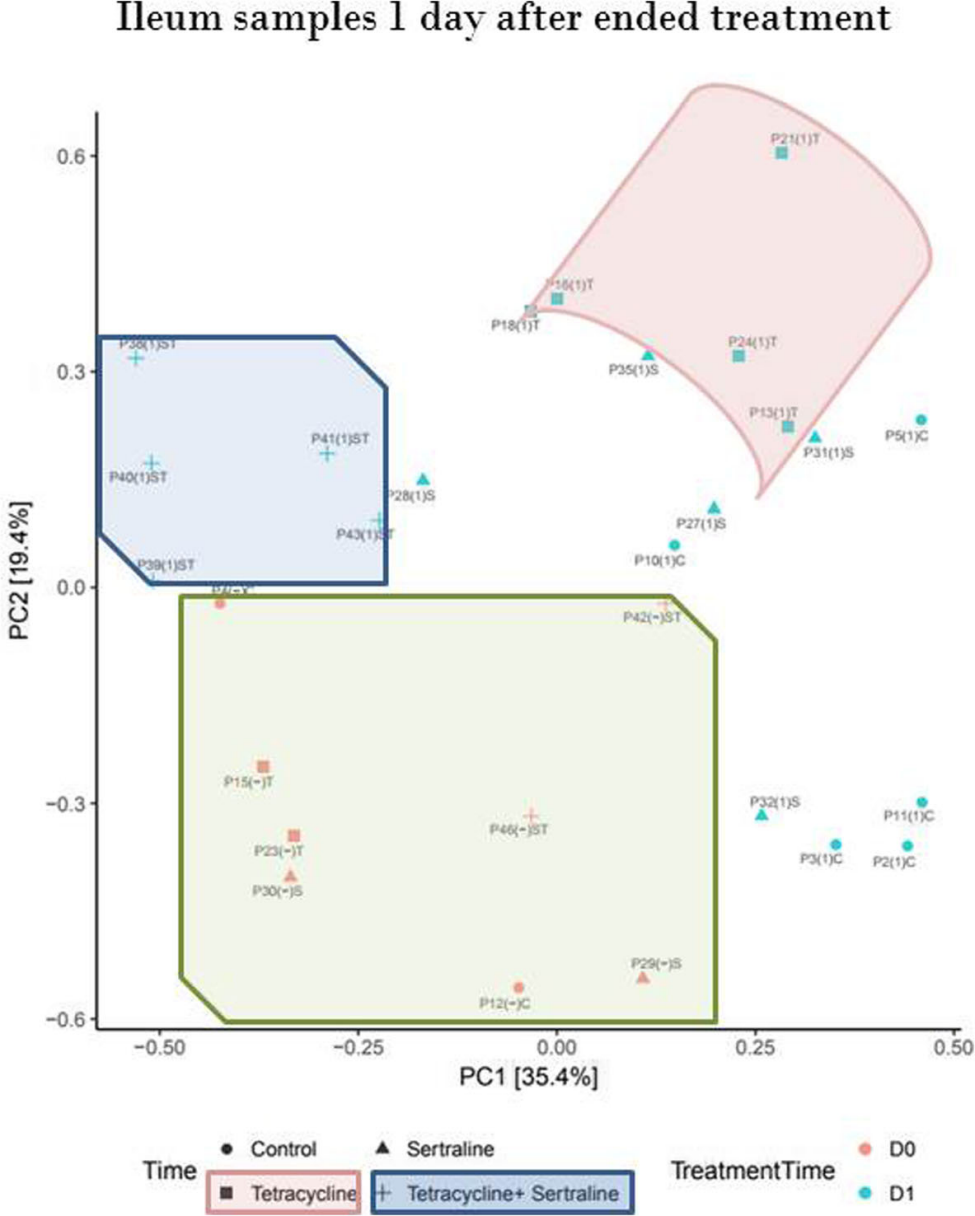
The 10 most abundant families in ileum samples obtained at time T[0], T[7] and T[15] The 10 most abundant genera in percent. Each has both a broad name (Phylum) and a specific name (Family). Samples were obtained from each treatment just prior to treatment start (D0/T[0]), 48 h after last day of treatment (D1/T[7]) and nine days after last day of treatment (D9/T[15])

Ileum samples were obtained from two, five and five pigs at time points T[0], T[7] and T[15], respectively. Before treatment start, the samples had a large diversity in microbiota composition (Fig. 3). At time T[7] a treatment-associated clustering of ileum microbiota samples from tetracycline- and sertraline/tetracycline treated groups was observed, while there were no distinct clustering of ileum samples from pigs in either control or sertraline treated groups (Fig. 3). For samples obtained at time T[7] the abundance of the Lactobacillaceae had the most distinct differences between groups. The level of the Lactobacillaceae had a significantly lower abundance in the tetracycline and sertraline/tetracycline treated groups compared to the level in the samples from the un-medicated control pigs. Furthermore, the abundance of the Lactobacillaceae was significantly lower in the sertraline/tetracycline treated group compared to samples from the tetracycline- or sertraline-treated groups. In contrast, the abundance of the Streptococcaceae in the control group was significantly higher than the abundance of Streptococcaceae in the tetracycline and sertraline/tetracycline treated groups. Furthermore, the abundances of Streptococcaceae were significantly lower in the sertraline/tetracycline compared to samples from the tetracycline and sertraline treated groups (Fig. 4, Additional file 2: Figure S2). The average abundance of Enterobacteriaceae in ileal samples from the tetracycline-treated group had a significantly higher average abundance of Enterobacteriaceae compared to any of three other groups (Fig. 4). For the remaining seven out of the ten overall most abundant families in the ileum samples (Fig. 4), there were no significant differences in the abundances between groups. At time point T[15], there were no longer any significant differences in the abundances of the 10 most abundant families across all ileal samples between the different treatment groups (Fig. 4), and no treatment-associated clustering of the ileum derived microbiota-samples (Additional file 3: Figure S1).

### Average daily weight gain between groups

The average daily weight gain per pig per group varied between 0.4–0.8 g/day. There was no statistical difference between the groups in average daily weight gain per group.

## Discussion

The main purpose of the study was to investigate if sertraline would re-sensitize an intestinal tetracycline-resistant population of bacteria, in particularly coliforms, to tetracycline in piglets. Secondly, the question as to how oral treatment with sertraline with or without tetracycline may impact the gut microbiota composition in relation to treatment and nine days after withdraw of medication, was studied.

Initially, there are some methodic issues of the study to address; First, as regulation of the European Union does not allow tetracyclines, or any other antibiotics, as in-feed growth promoters [14], the use of tetracycline in agriculture within EU is always related to a treatment-related aspect. In pig production tetracycline is the most commonly used antibiotic for treatment of enteric diseases [1]. Therefore, it could be argued if an experimental diarrhea model in pigs would have been more appropriate to address the question of helper-drug activity of sertraline. Such model is, however, fairly difficult to establish experimentally with success [15]. Often, only a limited amount of the pigs would develop diarrhea, likely because porcine enteritis is a complex disease with several factors contributing to diarrhea development [16]. Secondly, since all pigs were initially colonized with tetracycline resistant bacteria before inoculation with the indicator *E. coli* bacteria, this step could perhaps have been omitted. However, to ensure that each pig would have an equal challenge of a marked tetracycline-resistant bacteria, in which sertraline and tetracycline synergy had been confirmed in vitro [10], the inoculation step remained in the experimental design. Thirdly, the concentration of sertraline used to obtain in vitro synergy with tetracycline, is considerable higher than the maximal (human) plasma concentrations of sertraline (0.25 mg/L) [17], and therefore negative in vivo results may have been expected. However, to the authors’ knowledge there is no literature available on the concentration of sertraline in intestine of either animals or humans after oral medication. In the present study, we used daily doses of sertraline that would correspond to the maximum dose given to a person with severe mental disorders e.g. severe anxiety. Higher doses of sertraline may cause a general immune suppression, which increase the risk of infections [18]. Finally, even though tetracycline are used less frequently in human medicine than in agriculture, the high carriage of commensal tetracycline resistant bacteria in food-producing animals do possess a public risk, in particular because tetracycline resistance is likely to co-select for other, more human relevant antibiotics [19], therefore we find it relevant to investigate synergy of sertraline/tetracycline on the tetracycline-resistant commensal microbiota in pigs. Based on these considerations, we find the experimental design and treatment doses used appropriate to address the research questions raised above.

The heavily fecal shedding of tetracycline resistant bacteria in all pigs before the experimental inoculation (Fig. 1) is most likely caused by the intensive use of antibiotics in the pig industry in the last decades, as at a population level there is a strong association between the use of antibiotics and the proportion of bacteria resistant to antibiotics [5, 20, 21].

For all treatment groups, the treatments were tolerated well by the pigs with no clinical signs despite that a high-dose sertraline were administered to two of the treatment groups (Group 2 and 4). Toleration of the clinical relevant concentrations of the helper-drugs by the animals (or humans) is probably underrated [13] e.g. in another recent published study evaluating in vivo helper-compound activity of thioridazine, also a neurotrophic active medical compound, the authors report that their study had to pre-terminate due to severe side-effects of thioridazine in pigs with the concentrations used [22].

As expected, the pigs did not show clinical affection of the high-dose inoculum indictor bacteria. Fecal shedding of the indicator bacteria was very high two days after inoculation (Fig. 2), and a part of the inoculum there has probably mainly transited the intestinal tract with a only minor degree of colonization in ileum at T[0] (Fig. 2). For the tetracycline and sertraline/treated groups the fecal shedding of the indicator bacteria decreased significantly from time T[7] to T[15], while level in ileum remained at the same level as the level at T[0] for all groups (Fig. 2) indicating that some degree of intestinal colonization had occurred. For the tetracycline treated group, but not the sertraline/tetracycline, there was a significant increase in the intestinal level of the tetracycline resistant indicator *E. coli* strain shortly after withdrawal of tetracycline treatment (Fig. 2). This could indicate that sertraline may counteract the selective pressure of tetracycline, however, the level of the indicator *E. coli* remained as high as the average level of the indicator bacteria in ileal samples from the un-medicated control (3.8 log_10_ CFU) (Fig. 2). Furthermore, within the group of sertraline/tetracycline treated pigs, there was a rise in the number of total tetracycline resistant coliform bacteria at T[7] compared to control group (Fig. 1), hence, the suggested counteracting of a selective pressure, indicated by the lower level of resistant bacteria in the sertraline/tetracycline compared to tetracycline group, is not likely to have clinical impact. The difference between the impact of sertraline/tetracycline on the level of the indicator *E. coli* bacteria and the lack of impact on total tetracycline resistant coliforms could possibly be explained by the various types of tetracycline resistance [23], of which tetracycline and sertraline synergy has only been documented for the efflux-mediated type of tetracycline resistance, a mode of action present in the indicator *E. coli* strain [10].

The results on the selective pressure on the indicator bacteria is in contrast with the observations on the total tetracycline resistant coliforms at T[7], in the fecal samples. The fecal samples from tetracycline and sertraline/tetracycline treated groups had similar levels of tetracycline resistant coliforms, and the level tetracycline resistant coliforms of these two groups was significantly higher than the control group level at T[7] (Fig. 1). Similarily, theof intestinal tetracycline resistant coliforms was also significantly higher in the tetracycline treated group compared to the control group (Fig. 1). The finding of tetracycline treatment may select for a higher level of resistant bacteria followed by a return to the level before medication in a time span (in our study, nine days) from last day of short-time antibiotic treatment (Fig. 1)) is in accordance with previous observations [24]. However, nine days after ended treatment both the tetracycline and sertraline/tetracycline treated groups had levels of fecal tetracycline resistant coliform comparable to the un-medicated control group, implying that sertraline do not potentiate the effect of tetracycline in a population with a high proportion of commensal, tetracycline resistant bacteria. For the intestinal samples, however, the tetracycline treated group was the only group in which the level of tetracycline resistant coliforms remained significantly higher nine days after treatment compare to the level before treatment for the same group (Fig. 1). The level of tetracycline resistant coliforms were, nevertheless, not higher in the tetracycline group at nine days after ended treatment compared to the other treatment/control groups. Taken together, these results of the present study confirm a recent study by Graesboll et al. [24] reporting that five days treatment with tetracycline may transiently result in increased total count and proportion of tetracycline resistant coliform (Fig. 1).

In agreement with the significantly increased level of tetracycline resistant bacteria right after ended treatment for tetracycline (+/− sertraline) treated groups, treatment-associated grouping of ileal microbiota samples of pigs from Group 2 (tetracycline treated) and Group 4 (sertraline/tetracycline treated) were found (Fig. 3). Samples from Group 2 and 4 assembled individually, while samples from un-mediated control pigs and sertraline treated pigs did not demonstrate treatment-associated microbiota uniformities. The different compositions of microbiotas indicate that sertraline combined with tetracycline *has* an interactive effect that is different from the impact of the sum of changes caused by individual treatment with either sertraline or tetracycline, proposing that there might be some synergy or at least interaction between tetracycline and sertraline on the microbiota composition in vivo.

Nine days after ended treatment there were no indication of treatment-associated clustering of ileum-derived microbiotas (Additional file 3: Figure S1), corresponding to the observations done on comparisons of CFU counts from the different treatment groups.

The amount on literature concerning the microbiota of animals as humans has increased almost exponentially the last decade. Yet, *the* optimal intestinal microbiota composition is yet to be described. Nevertheless, a recent publication combining metagenomics and microbial function genetics and the impact of in-feed antibiotics has enlighten the understanding on how a shift in bacterial population may also shift energy production and conversion and, hence, growth of the pigs. In the above mentioned study, feeding pigs with a commercial available (in the US) performance-enhancing mix of antibiotics lead to a bacterial shift, dominated by an increase in phylum *Proteobacteria*, in particular *E. coli*, in the medicated pig compared to the un-medicated control [25]. These observations are in agreement with the present study, in which a significant increase in the abundance of *E. coli* in ileal samples from the tetracycline-treated pigs compared with ileum samples from the un-treated control pig (Additional file :3 Figure S1). The unique increase in the abundance of *E. coli* in the tetracycline treated group at time point T[7] is in agreement with the unique increase of the CFU per gram of indicator *E. coli* at the same time point. The same increases in either abundance or CFU count of *E. coli* were, however, not evident in the ileum samples from the pigs treated with tetracycline in combination with sertraline (Fig. 1 Fig. 4). Rather, for the latter mentioned group the level of the family Streptococcaece was significantly increased, while the abundances of the family of Lactobacillaceae was dramatically decreased at time point T[7]. *Lactobacillus* spp. are probably the most treasured bacteria in term of general health-promoting probiotic effects [26], and hence a low level of *lactobacillus* would be considered strongly un-favorable. It can be concluded that sertraline and tetracycline have an interaction that create a measurable response on CFU per gram and microbiota composition right after ended treatment, although the effect may neither be major (in terms of decreasing the level of tetracycline resistant coliforms) nor beneficial (in terms of an optimal microbiota composition). However, in accordance with the observations done on the cultivable bacteria, the microbiota compositions nine days after withdrawal of treatment did no longer show any association with any previous treatments (Fig. 4, Additional file 2: Figure S1), underling that the five days of treatment with tetracycline in therapeutic concentrations (+/− sertraline) did not have long-lasting effects on the intestinal bacterial population, including the tetracycline resistant population.

## Conclusion

The high carriage of antimicrobial resistance bacteria in the porcine autochthonous microbiota calls for new treatment strategies to ensure continuous treatment success and human food safety. In this study, the impact of sertraline as a tetracycline helper-compound had only very limited effect and is not likely to have a clinical importance. Furthermore, the increased level of tetracycline-resistant coliforms in the tetracycline-medicated group (without sertraline added) shortly after ended treatment returned to a level comparable to the un-medicated control group in less than two weeks after ended treatment. Similarly, the composition of ileal microbiota from tetracycline and tetracycline/sertraline medicated groups, clustered according to treatment only immediately after treatment. Nine days after ended treatment no treatment-associated effect could be detected.

In conclusion under the conditions tested there were no beneficial effect of sertraline as a tetracycline helper-compound, despite the previously reported promising in vitro synergy between tetracycline and sertraline.

Nevertheless, the carriage of high level of tetracycline-resistant bacteria among all of the commercial bought piglets used in the study, calls for more prudent antibiotic use in the pig production, new antibiotics or novel efficient helper-compound to re-sensitize bacteria to the traditional antibiotics.

## Methods

### Pigs and housing

Forty-eight healthy female Danish landrace piglets (seven weeks of age) with normal faecal consistency were bought from a commercial pig producer. Unfortunately, data on previous antibiotic treatment for each individual pig was not available. At arrival at the experimental animal unit of University of Copenhagen, Frederiksberg campus the pigs were weighted and randomly allocated to one of four groups, placed in four different pens. Hereafter, the pigs were allowed to acclimatize for one week before starting the trial. The pigs were kept on restricted diet of commercial feed (Svine Erantis Brogaarden ApS, Lynge, Denmark) according to age (550 g/pig/day) and had free access to tap water. The pigs were euthanized by an initially anesthetizing shoot with a captive bolt-pistol followed by incision on jugular vein and bleeding of the pigs. All procedures performed on the pigs were approved and licensed by the Danish Animal Experiments Inspectorate (license no. 2016-15-0201-01144).

### Experimental design

After one week of acclimatization and two days before treatment start (time point T[− 2]), all pigs were weighted and fecal samples were obtained from all 12 pigs in each of the four groups. Hereafter all pigs were orally inoculated with a tetracycline-resistant indicator strain of *E. coli* (strain details are given below) in 50 mL of water-diluted, blended, soft cat food (supermarket brand), which the pig found highly palatable. Inoculation was thus voluntary and stress-free for all pigs, which ensured that all pigs be equally and heavily colonized with a rifampicin-marked tetracycline resistant strain of *E. coli*. Two days after inoculation and just prior to treatment start (time point T[0]), all pigs were weighted and individual fecal samples were obtained. Two randomly chosen pigs from each group were hereafter euthanized, and ileum samples were obtained from these eight pigs to represent the ileal microbiota composition before treatment. At the same day, the pigs in Group 2 (tetracycline treated), Group 3 (sertraline treated) and Group 4 (sertraline/ tetracycline treated) received individual, voluntary oral treatment (sertraline or compounds dissolved in blended cat food) for five consecutive days (Doxylin© (Dopharma Research, Raamsdonksveer, Netherlands) (24 mg/kg body weight corresponding to 12.5 mg doxycycline (a tetracycline antibiotic)/kg body weight; Sertrone© (KRKA Sverige, Stockholm, Sweden) (one 100 mg sertraline-containing tablet daily, corresponding to approximately four mg/kg body weight). Pigs in Group 1 remained un-medicated, but received the same amount (10 ml) of the water-diluted blended cat food. During the treatment period, pigs in Group 2 and 4 were weighted each day to adjust the amount of Doxylin per pig per kg body weight per day. Forty-eight hours after ended treatment period (time point T[7]), fecal samples were obtained from all pigs and hereafter five pigs in each group were chosen randomly and euthanized followed by obtaining of ileum samples from all euthanized pigs. The remaining five pigs in each group remained un-medicated the following nine days post treatment. Nine days after ended treatment (time point T[15]), individual fecal samples were obtained, pigs were euthanized and ileum samples were collected from all pigs as well. All pigs were clinically healthy throughout the study period.

Fecal samples were obtained by either spontaneously defecation without the sample reaching the floor or obtained directly from rectum. All samples were collected with sterile latex gloves.

Ileum samples were obtained by localising the ostium ileocecale and hereafter measuring five cm in the oral direction. At this location a sterile pean was placed to occlude the intestinal lumen. Another 10 cm in the oral direction were measured and a second occlusive pean placed to allow resection of the ileal segment. The section was placed on a disinfected table and cut open to expose the mucosal surface. A sterile glass microscope slide was used to scrape five times into the depth of the mucosal layer. All material hereof, i.e. the mucosal tissue and intestinal content, was transferred to a sterile petri dish.

All samples were processed (serial dilution plating or DNA extraction) within the same day the samples had been collected.

### Preparation of inoculum of indicator bacteria

The *E. coli* tetracycline resistant “*E. coli* O2” was chosen as an indicator strain because in vitro synergy with sertraline has been documented and investigated in detail [10]*.* Secondly, the strain does not contain any of the primary virulence factors associated with porcine enterotoxigenic *E. coli* (toxins, fimbria F4/F18 ect.) [27]*.* The strain contains two large plasmids; one encoding resistances toward seven different antimicrobials, including tetracycline [28], and a virulence plasmid [29], genes encoding for increased survival in extra-intestinal compartments, e.g. the human urinary tract system [30]. For re-isolation purposes, rifampicin resistance was induced by standard procedures*.* The rifampicin mutant strain was confirmed to have the same tetracycline resistance properties and synergy with tetracycline as previously reported for the wild-type strain of *E. coli* O2 [10].

The strain had been stored at − 80 °C in Brain and Heart Infusion (BHI) broth (Oxoid, Basingstoke, UK) in 15% (*v*/v) glycerol until needed. The day before inoculation four colonies of the strain were picked from an agar plate (Oxoid, CM0055) supplemented with 5% calf blood. Each colony was inoculated into a flask containing 250 mL BHI broth and incubated at 37 °C without shaking for 24 h to reach a concentration of approximately 10^9^ CFU. The four overnight grown cultures were pooled to one culture in a sterile Blue cap bottle (Sigma-Aldrich, Copenhagen, Denmark) and distributed into 50 mL centrifuge tubes each containing 40 mL of the pooled culture and centrifuged at 4 °C at 3000G for 15 min. Subsequently, the supernatant in each tube was discharged and the pellet re-suspended in 10 ml phosphate buffered solution (PBS), and again pooled together in one pool, of which each pig orally received five ml. To determine the exact dose administered to each pig, 100 μl of the pooled, re-suspended bacterial solution was used to make a 10-fold serially dilutions until 10^− 11^ dilution and from each dilution, 100 μl was plated on MH ager (Oxoid Ltd., Thermo Scientific, Roskilde, Denmark) to determine the final inoculation dose of 2 × 10^12^ CFU per pig.

### Bacterial quantification

Approximately one gram of each sample (fecal or ileum was diluted 1:10 in PBS in a Biomaster 80© filter bag (Stomacher, Seward Inc., Port Saint Lucie, FL, USA) and homogenized in a Stomacher machine (Stomacher, Seward Inc) for 1 min. Subsequently, serial 10-fold dilutions were made in 0.9% NaCl solution. From all dilutions (10^−1^to 10^− 7^), 10 μl were spotted on agar plates containing the following two media: MacConkey agar supplemented with 8 mg/L tetracycline (for enumeration of total tetracycline resistant coliforms), and MacConkey agar supplemented with 8 mg/L tetracycline, 50 mg/L ampicillin (Sigma-Aldrich) and 25 mg/liter rifampicin (Sigma-Aldrich) (For enumeration of the indicator strain *E. coli*).

*E. coli* K-12 MG1655 (tetracycline sensitive) and *E. coli* NCTC 50078 (tetracycline resistant) were included as control strains on all plates. For MacConkey agar supplemented with tetracycline, rifampicin and ampicillin, the inoculum strain (*E. coli*_O2 [31]) was included as well.

All serial dilutions were done and plated in duplicates. The plates were aerobically incubated for 24 h at 37 °C followed by colony enumeration.

For each plate, a count, expressed as the number of CFU per gram, was determined using a weighed arithmetic mean based on the two highest dilutions showing the separation between colonies, and finally, the number of CFU per gram was log10 transformed. To avoid exclusion of samples with a CFU per gram equaling zero from the analysis (as 0 cannot be log_10_ transformed), all numbers of CFU per gram sample, was added a constant of 1. This constant was chosen because log10 to 1 equals 0.

### PCR confirmation of re-isolation of inoculated indicator strain *E. coli*_O2

To verify that the isolates from fecal or ileal samples growing on the agar plate containing MacConkey supplemented with tetracycline, rifampicin and ampicillin plate were identical to the inoculum strain of *E. coli*, two randomly chosen isolates from each pig were confirmed as identical to the inoculum strain by an *E. coli*_O2 specific PCR as described in Kromann et al. (2017) [32].

### DNA extraction of fecal and ileum samples

Total DNA from 48 ileal and10 fecal samples, respectively, were extracted for determination of the microbiota composition. From the 10^− 1^ diluted homogenates used for the enumeration of bacteria, 800 μl homogenate from each of the samples was transferred to a 2 mL FastPrep© Lysis matrix E tube (MP Biomedical, Solon, OH, USA) and further homogenized on a FastPrep-24 Instrument (MP Biomedical) at 6 m/sec for 40 s, followed by centrifugation for 15 min at 10000G. Subsequently, 400 μl of the supernatant of each sample were processed on a Maxwell© RSC instrument (Promega Corporation, Mannheim, Germany) applying the RSC PureFood GMO kit (Cat. # AS1600) according to manufacturer’s protocol for extraction of DNA.

DNA concentrations were measured on an Agilent 2100 Bioanalyzer (Agilent Technologies, Waldbronn, Germany) before 16S rRNA gene amplicon library preparation.

### 16S rRNA gene amplicon library preparation

Bacterial V3–4 16S rRNA gene sequencing libraries of extracted DNA from 48 ileal samples and 10 fecal samples were prepared by a custom protocol based on an Illumina (Illumina, San Diego, CA, USA) protocol [33] according to previously done by Olsen et al. 2017 [34]. Briefly, for PCR amplification of the 16S rRNA gene fragments, 10 ng of extracted DNA was used as template. Each PCR reaction (25 μL) contained dNTPs (100 μM of each), MgSO4 (1.5 mM), Platinum® Taq DNA polymerase HF (2 mU), 1X Platinum® High Fidelity buffer (Thermo Fisher Scientific, USA) and tailed primer mix (400 nM of each forward and reverse). PCR conditions included: An nitial denaturation at 95 °C for 2 min, 35 cycles of amplification (95 °C for 20 s, 50 °C for 30 s, 72 °C for 60 s) and a final elongation at 72 °C for 5 min. Duplicate PCR reactions was performed for each sample and the duplicates were pooled after PCR. The forward and reverse tailed primers were designed according to Vo and Jedlicka [33] and contain a primer parts targeting the respective 16S rRNA gene fragments. Bacteria V3–4 [35]: 5’-CCTACGGGNGGCWGCAG (341F) and 5’-GACTACHVGGGTATCTAATCC (805R). The primer tails enable attachment of Illumina Nextera adaptors for sequencing in a subsequent PCR. The amplicon libraries were purified using Agencourt Ampure XP Bead (Beckman Coulter) using vendor recommended protocol, using a bead to sample ratio of 4:5 and the DNA was eluted in 33 μL of nuclease free water (Qiagen, Hilden, Germany). DNA concentration was measured using Quant-iT DNA Assay Kit, high sensitivity (Thermo Fisher Scientific, Roskilde, Denmark). Sequencing libraries were prepared from the purified amplicon libraries using a second PCR. Each PCR reaction (25 μL) contained 1x PCRBIO HiFi buffer (PCR Biosystems Ldt, London, UK), PCRBIO HiFi Polymerase (1 U) (PCR Biosystems Ldt), adaptor mix (400 nM of each forward and reverse) and 2 μL of amplicon library template. PCR was run with the following program: Initial denaturation at 95 °C for 2 min, 8 cycles of amplification (95 °C for 20 s, 55 °C for 30 s, 72 °C for 60 s) and a final elongation at 72 °C for 5 min. The sequencing libraries were purified using Agencourt Ampure XP Bead (Beckman Coulter, Redlands, CA, USA) using vendor recommended protocol, using a sample/bead ratio of 5:4 and the DNA was eluted in 20 μL of nuclease free water (Qiagen). DNA concentration was measured using Quant-iT DNA Assay Kit, high sensitivity (Thermo Fisher Scientific). Gel electrophoresis using Tapestation 2200 and D1000 High Sensitivity screentapes (Agilent Technologies) was used to check the product size and purity of randomly picked sequencing libraries.

### DNA sequencing

The purified sequencing libraries were pooled in equimolar concentrations and diluted to 4 nM. The samples were paired end sequenced (2x301bp) on a MiSeq (Illumina) using a MiSeq Reagent kit v3, 600 cycles (Illumina) following the standard guidelines for preparing and loading samples on the MiSeq. 20% Phix control library was spiked in to overcome low complexity issue often observed with amplicon samples.

### 16S rRNA gene amplicon bioinformatic processing

Forward and reverse reads were trimmed for quality using the software Trimmomatic v. 0.32 [36] with the settings SLIDINGWINDOW:5:3 and MINLEN:275. The trimmed forward and reverse reads were merged using the program FLASH v. 1.2.7 [37], with the settings -m 25 -M 200. The merged reads were dereplicated and formatted for use in the UPARSE workflow [38]. The dereplicated reads were clustered, using the usearch v. 7.0.1090 -cluster_otus command with default settings. Operational Taxanomic Units (OUT) abundances were estimated using the usearch v. 7.0.1090 -usearch_global command with -id 0.97. Taxonomy was assigned using the RDP classifier [39] as implemented in the parallel_assign_taxonomy_rdp.py script in QIIME [40], using the MiDAS database v.1.23 [41]. The results were analysed in R [42] through the Rstudio IDE using the ampvis package v.2.0 [43].

### Average weight gain

The average weight gain per pig was calculated as difference in body weight from the day of arrival to the experimental unit compared to the body weight of the pig at the day of euthanization divided by the days spend at the experimental unit.

### Statistical analysis

Statistical analyses were done using the software Graphpad Prism version 7 (Graphpad Software, Inc., La Jolla, CA, USA). At each time sampling point, differences between groups for log10 CFU on the five different types of agar plates, proportions of CFU counts, and abundances of different genera in the microbiota or average weight gain/group were analysed by one-way ANOVA. In addition, differences within each group at different sampling times were analysed in the same manner. The one-way ANOVA was followed by Turkey’s t-test for multiple comparisons. A statistical difference of *P* < 0.5 was considered statistically significant.

## Additional files

Additional file 1: **Table S1.** Sample concentration and number of reads after sequencing. “Reads” is the number of reads after sequencing after sequencing, quality control and bioinformatics processing, “Observed” is the number of observed operational taxonomic units in 10000 reads, while Shannon is the Shannon index observed in 10000 reads. (DOCX 19 kb)

Additional file 2: **Figure S2.** The 25 most abundant families genera. The overall 25 most abundant genera in percent. Each has both a broad name (Phylum) and a specific name (Genus). Samples from the untreated control group ©, sertraline treated (S), tetracycline (T) and sertraline/tetracycline treated groups were obtained (ST) (D0/T[0]), 48 h after last day of treatment (D1/T[7]) and nine days after last day of treatment (D9/T[15]). Feces samples were only obtained nine days after ended treatment just prior to treatment start. From the latter two, samples P01, P06, P07, P08 and P09 were obtained from pigs in the un-medicated control group, while samples P14, P17, P19, P20 and P22 were obtained from pigs that had received tetracycline treatment. (JPG 149 kb)

Additional file 3: **Figure S1.** Principle component analysis (PCA) of ileum microbiota composition at time T[0] and T[15]. Identification of samples with similar microbial communities using multivariate statistics (PCA). Each red or blue point represent the microbita composition in a specific sample obtained just prior to treatment start (D0/T[0]) or nine days after the last day of treatment (D9/T[15]), respectively. (JPG 42 kb)

## Abbreviations

CFU: Colony forming units
OUT: Operational Taxanomic Units
PCR: Polymerase Chain Reaction
SSRI: Selective Serotonin Reuptake Inhibitor
