# Supplementary material for: In vitro synergy of sertraline and tetracycline cannot be reproduced in pigs orally challenged with a tetracycline resistant Escherichia coli
Source: BMC Microbiol. 2019 Jan 11;19:12. doi: 10.1186/s12866-018-1383-5 (PMC6330422; doi:10.1186/s12866-018-1383-5)
Supplement: Supplementary file 1 — Table S1. Sample concentration and number of reads after sequencing. “Reads” is the number of reads after sequencing after sequencing, quality control and bioinformatics processing, “Observed” is the number of observed operational taxonomic units in 10000 reads, while Shannon is the Shannon index observed in 10000 reads. (DOCX 19 kb) [file 12866_2018_1383_MOESM1_ESM.docx]

**Table S1**. **Sample concentration and number of reads after sequencing**. “**Reads”** is the number of reads after sequencing after sequencing, quality control and bioinformatics processing, “**Observed**” is the number of observed operational taxonomic units in 10000 reads, while **Shannon** is the Shannon index observed in 10000 reads.

| **Sample name** | **Treatment group** | **Sampling site** | **Sampling time** | **Extraction concentration**  **(ng/µl)** | **Library concentration**  **(ng/µl)** | **Reads** | **Observed** | **Shannon** |
| --- | --- | --- | --- | --- | --- | --- | --- | --- |
| P12(-)C | Control | Ileum | T[0] | 824 | 7.9 | 22016 | 52 | 2.0 |
| P4(-)C | Control | Ileum | T[0] | 831 | 1.7 | 19293 | 70 | 1.6 |
| P23(-)T | Tetracycline | Ileum | T[0] | 475 | 6.8 | 18696 | 49 | 1.5 |
| P15(-)T | Tetracycline | Ileum | T[0] | 349 | 1.2 | 19077 | 71 | 1.8 |
| P30(-)S | Sertraline | Ileum | T[0] | 641 | 7.2 | 19249 | 58 | 1.9 |
| P29(-)S | Sertraline | Ileum | T[0] | 651 | 9.9 | 24648 | 40 | 1.3 |
| P46(-)ST | Sertraline/tetracycline | Ileum | T[0] | 768 | 4.7 | 14986 | 61 | 2.1 |
| P42(-)ST | Sertraline/tetracycline | Ileum | T[0] | 409 | 6 | 18171 | 45 | 1.6 |
|  |  |  |  |  |  |  |  |  |
| P11(1)C | Control | Ileum | T[1] | 874 | 5 | 9858 | 45 | 1.1 |
| P2(1)C | Control | Ileum | T[1] | 486 | 14 | 27668 | 66 | 1.4 |
| P10(1)C | Control | Ileum | T[1] | 898 | 0.2 | 2432 | 57 | 2.8 |
| P5(1)C | Control | Ileum | T[1] | 849 | 9 | 21804 | 81 | 1.7 |
| P3(1)C | Control | Ileum | T[1] | 349 | 15.2 | 26516 | 83 | 1.3 |
| P18(1)T | Tetracycline | Ileum | T[1] | 101 | 12.3 | 27176 | 59 | 2.0 |
| P24(1)T | Tetracycline | Ileum | T[1] | 276 | 17.3 | 24395 | 110 | 1.7 |
| P16(1)T | Tetracycline | Ileum | T[1] | 228 | 3.8 | 20571 | 88 | 1.7 |
| P13(1)T | Tetracycline | Ileum | T[1] | 449 | 10.8 | 24951 | 67 | 1.8 |
| P21(1)T | Tetracycline | Ileum | T[1] | 796 | 14.2 | 21591 | 48 | 1.5 |
| P28(1)S | Sertraline | Ileum | T[1] | 512 | 12.3 | 23920 | 76 | 2.6 |
| P35(1)S | Sertraline | Ileum | T[1] | 760 | 17.1 | 20396 | 72 | 2.1 |
| P32(1)S | Sertraline | Ileum | T[1] | 38 | 9.4 | 21216 | 73 | 2.1 |
| P31(1)S | Sertraline | Ileum | T[1] | 522 | 15.2 | 20055 | 72 | 1.9 |
| P27(1)S | Sertraline | Ileum | T[1] | 973 | 15 | 18846 | 43 | 2.4 |
| P38(1)ST | Sertraline/tetracycline | Ileum | T[1] | 352 | 15.6 | 6265 | 62 | 0.9 |
| P39(1)ST | Sertraline/tetracycline | Ileum | T[1] | 471 | 4.7 | 22028 | 355 | 2.5 |
| P41(1)ST | Sertraline/tetracycline | Ileum | T[1] | 480 | 15 | 19725 | 67 | 4.1 |
| P40(1)ST | Sertraline/tetracycline | Ileum | T[1] | 408 | 12 | 22084 | 90 | 1.1 |
| P43(1)ST | Sertraline/tetracycline | Ileum | T[1] | 394 | 8.1 | 19725 | 65 | 1.0 |
|  |  |  |  |  |  |  |  |  |
| P1(9)C | Control | Ileum | T[9] | 361 | 4.7 | 16526 | 94 | 2.5 |
| P6(9)C | Control | Ileum | T[9] | 388 | 9 | 21831 | 238 | 4.6 |
| P7(9)C | Control | Ileum | T[9] | 21 | 8.1 | 17193 | 99 | 2.4 |
| P8(9)C | Control | Ileum | T[9] | 40 | 1.9 | 22983 | 104 | 2.6 |
| P9(9)C | Control | Ileum | T[9] | 5 | 1.1 | 17685 | 79 | 2.0 |
| P14(9)T | Tetracycline | Ileum | T[9] | 269 | 1.4 | 17804 | 143 | 2.6 |
| P17(9)T | Tetracycline | Ileum | T[9] | 6 | 1.4 | 16916 | 83 | 2.1 |
| P19(9)T | Tetracycline | Ileum | T[9] | 167 | 0.4 | 6961 | 72 | 1.3 |
| P20(9)T | Tetracycline | Ileum | T[9] | 17 | 2.8 | 21957 | 66 | 1.8 |
| P22(9)T | Tetracycline | Ileum | T[9] | 443 | 8 | 22013 | 54 | 1.3 |
| P25(9)S | Sertraline | Ileum | T[9] | 73 | 10.3 | 20810 | 95 | 1.8 |
| P26(9)S | Sertraline | Ileum | T[9] | 21 | 4.6 | 21520 | 125 | 0.8 |
| P33(9)S | Sertraline | Ileum | T[9] | 298 | 9.8 | 23197 | 83 | 1.2 |
| P34(9)S | Sertraline | Ileum | T[9] | 86 | 12.5 | 22252 | 197 | 1.9 |
| P36(9)S | Sertraline | Ileum | T[9] | 182 | 2 | 18419 | 69 | 1.9 |
| P37(9)ST | Sertraline/tetracycline | Ileum | T[9] | 401 | 5.8 | 21941 | 84 | 1.8 |
| P44(9)ST | Sertraline/tetracycline | Ileum | T[9] | 55 | 7.3 | 21290 | 157 | 2.4 |
| P45(9)ST | Sertraline/tetracycline | Ileum | T[9] | 509 | 4.6 | 20903 | 99 | 2.0 |
| P48(9)ST | Sertraline/tetracycline | Ileum | T[9] | 106 | 5 | 18583 | 186 | 2.1 |
| P50(9)ST | Sertraline/tetracycline | Ileum | T[9] | 264 | 7.9 | 21538 | 364 | 2.4 |
|  |  |  |  |  |  |  |  |  |
|  |  |  |  |  |  |  |  |  |
| P1-F | Control | Feces | T[9] | 5 | 5.7 | 15840 | 419 | 4.7 |
| P6-F | Control | Feces | T[9] | 4 | 9.6 | 23317 | 238 | 4.8 |
| P7-F | Control | Feces | T[9] | 2 | 6.1 | 20101 | 395 | 4.6 |
| P8-F | Control | Feces | T[9] | 2 | 8.8 | 21069 | 355 | 4.9 |
| P9-F | Control | Feces | T[9] | 5 | 10.1 | 19291 | 402 | 4.8 |
| P14-F | Tetracycline | Feces | T[9] | 7 | 11.3 | 19445 | 408 | 4.7 |
| P17-F | Tetracycline | Feces | T[9] | 3 | 5 | 16416 | 393 | 4.9 |
| P19-C |  |  |  |  |  |  |  |  |
| P20-C |  |  |  |  |  |  |  |  |
| P20-F | Tetracycline | Feces | T[9] | 3 | 8.2 | 17483 | 390 | 4.6 |
| P22-F | Tetracycline | Feces | T[9] | 3 | 11 | 20634 | 380 | 4.5 |
|  |  |  |  |  |  |  |  |  |
