# Supplementary figures and images for: In vitro synergy of sertraline and tetracycline cannot be reproduced in pigs orally challenged with a tetracycline resistant Escherichia coli
Source: BMC Microbiol. 2019 Jan 11;19:12. doi: 10.1186/s12866-018-1383-5 (PMC6330422; doi:10.1186/s12866-018-1383-5)

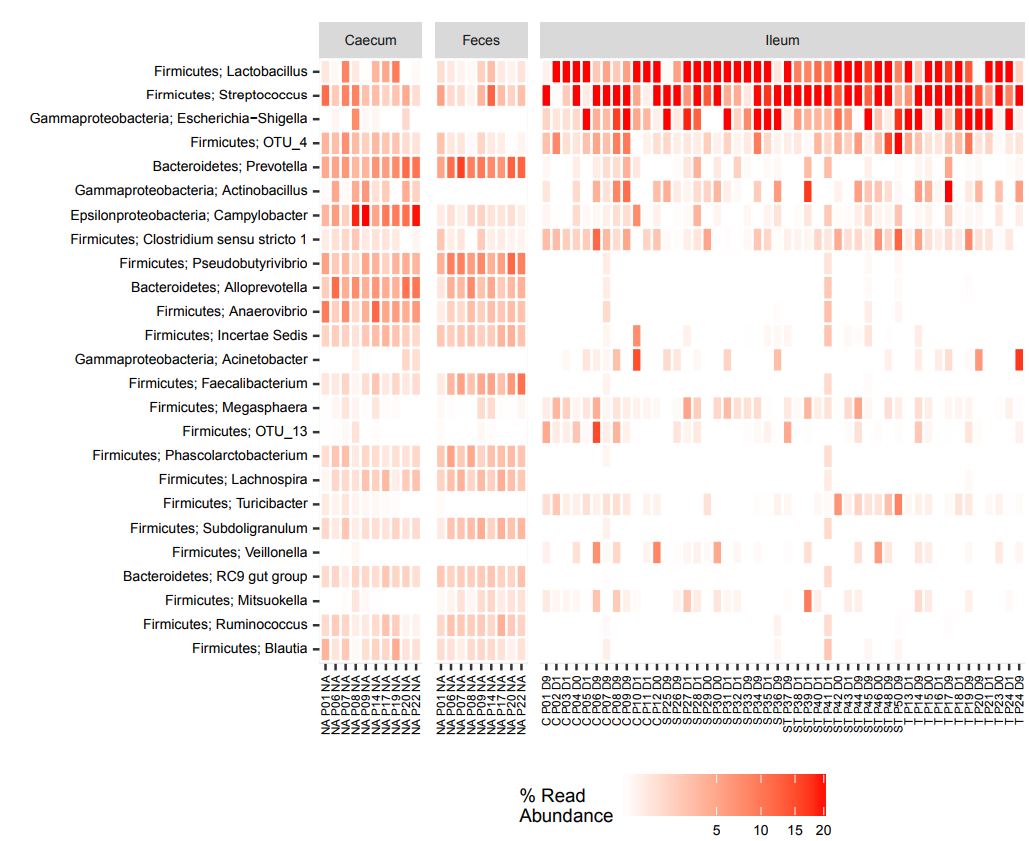

Supplement: Supplementary file 2 — Figure S2. The 25 most abundant families genera. The overall 25 most abundant genera in percent. Each has both a broad name (Phylum) and a specific name (Genus). Samples from the untreated control group ©, sertraline treated (S), tetracycline (T) and sertraline/tetracycline treated groups were obtained (ST) (D0/T[0]), 48 h after last day of treatment (D1/T[7]) and nine days after last day of treatment (D9/T[15]). Feces samples were only obtained nine days after ended treatment just prior to treatment start. From the latter two, samples P01, P06, P07, P08 and P09 were obtained from pigs in the un-medicated control group, while samples P14, P17, P19, P20 and P22 were obtained from pigs that had received tetracycline treatment. (JPG 149 kb) [file 12866_2018_1383_MOESM2_ESM.jpg]

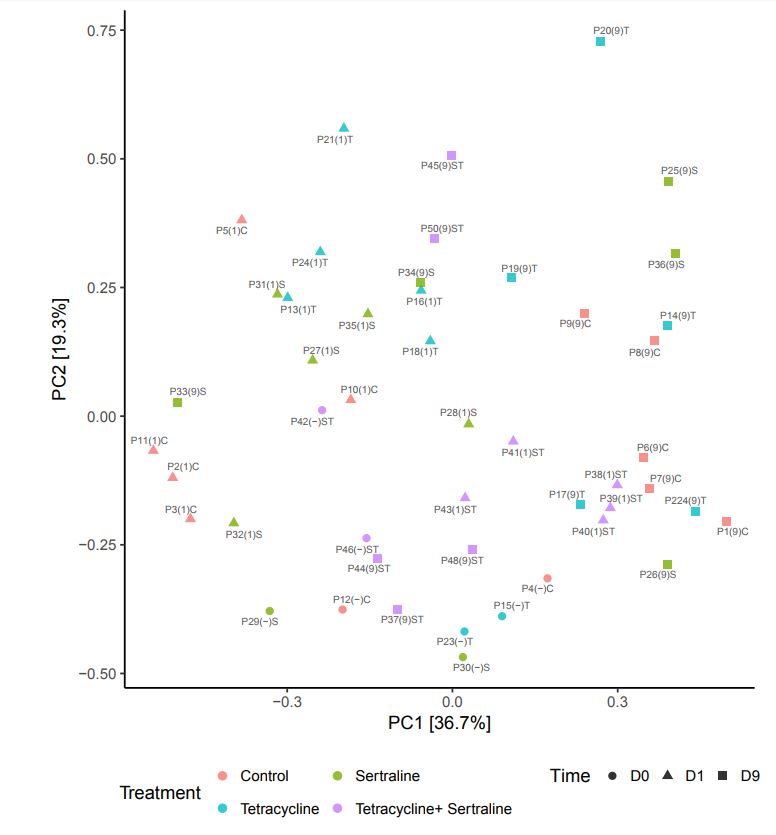

Supplement: Supplementary file 3 — Figure S1. Principle component analysis (PCA) of ileum microbiota composition at time T[0] and T[15]. Identification of samples with similar microbial communities using multivariate statistics (PCA). Each red or blue point represent the microbita composition in a specific sample obtained just prior to treatment start (D0/T[0]) or nine days after the last day of treatment (D9/T[15]), respectively. (JPG 42 kb) [file 12866_2018_1383_MOESM3_ESM.jpg]
